# Sundowning Syndrome in Dementia: Mechanisms, Diagnosis, and Treatment

**DOI:** 10.3390/jcm14041158

**Published:** 2025-02-11

**Authors:** Michalina Reimus, Mariusz Siemiński

**Affiliations:** 1 Emergency Department, University Clinical Centre, 80-214 Gdansk, Poland; mreimus@uck.gda.pl; 2 Department of Emergency Medicine, Medical University of Gdansk, 80-210 Gdansk, Poland

**Keywords:** sundowning syndrome, dementia, depression, insomnia

## Abstract

“Sundowning syndrome” refers to the evening decline in mental state among cognitively impaired patients. This phenomenon is well known, but it is not entirely understood. Its prevalence ranges from 1.6% to 66% of patients with dementia. Development of SS relies on neurodegeneration, the presence of sleep disorders, circadian rhythm of patients’ activities, and mood disorders. Therefore, patients with SS need very precise diagnostic workup aiming at defining the exact cause of the syndrome. Potential therapeutic modalities include behavioral and environmental interventions and pharmacological approaches. Pharmacotherapy with sedatives can by effective but is related to severe side effects. Behavioral interventions are more efficacious but require intense involvement of caregivers. This article discusses the biological processes that may underlie SS and proposes potential diagnostic procedures and therapeutic interventions.

## 1. Introduction

Sundowning syndrome (SS) is a term often used in the daily practice of physicians taking care of patients with neurodegenerative diseases to depict a pathological change in the patients’ behavior that appears in the evening or during the night. This change usually takes the form of agitation, aggression, anxiety, or delirium [1]. However, the name of this syndrome is not found in international classifications of diseases, and no scientific society has developed diagnostic criteria or procedural standards for this syndrome. The fact that SS, regardless of its clinical significance, is so neglected formally is not easy to explain. It is a clinical phenomenon of great significance and widely spread among patients with dementia, resulting in a serious burden for the caregivers. Its prevalence reaches over 60% in some studies and is an important reason for the placement of patients in nursing facilities. It is commonly considered as a part of a larger group of behavioral symptoms appearing secondarily to neurodegeneration; thus, not requiring separate classification. This does not change the fact that it is an important clinical phenomenon; hence, we decided to focus on that topic in the following narrative review. Herein, we aimed to present to the clinicians available data on the clinical picture, diagnostics, and therapeutic interventions in SS. This article presents the clinical features of SS in an attempt to conjure a definition and discusses possible mechanisms leading to its occurrence, principles for its diagnosis and diagnostic procedures, and possibilities for therapeutic interventions.

## 2. Definition of Sundowning Syndrome

Although clinicians often use the term “sundowning”, its understanding is based on intuition, as it does not have an unambiguous definition. Generally, this term refers to the occurrence or exacerbation of psychiatric symptoms in the evening hours in people with dementia. However, there are discrepancies in this definition, as some authors use this term to describe psychiatric symptoms that appear in the evening in elderly people, regardless of their state of cognitive functions [2].

It is also not clearly defined which psychiatric symptoms are part of the clinical picture of SS. Often, authors consider agitation, aggression, anxiety, wandering, disorientation, hallucinations, and delusions. Some authors are willing to include deterioration of cognitive functioning, e.g., memory impairment, in the clinical picture of SS [1].

The establishment of a time frame for the occurrence of SS also remains controversial. It is most often assumed that symptoms should appear in the late afternoon or evening. There are also proposals to use this term to refer to all psychiatric symptoms that appear at night. Another attempt to define this syndrome is as referral to psychiatric symptoms that simply develop in the dark [3].

There are also some researchers denying the justification for distinguishing a separate SS. Some authors postulate that these symptoms fall within the spectrum of dementia, which is no different from symptoms that occur at other times of the day, and that linking them specifically to the night period is only a consequence of the greater inconvenience to the people taking care of these patients when these symptoms appear after dark [1,3].

Scientists estimating the prevalence of SS must use a precise definition. A review of the diagnostic criteria used in recent epidemiological studies may present an approximation of how researchers understand this term. Shih et al. interpreted SS to be solely agitation that repeatedly appears in the evenings; therefore, they used the Cohen–Mansfield Agitation Inventory to detect and assess SS [4]. A specific questionnaire (the Sundown Syndrome Questionnaire, SSQ) was created and used in another study. The SSQ inquired about the presence of behavioral problems (e.g., aggression, disorientation, wandering, and anxiety), the time of day/night when they appear, and their frequency. A diagnosis of SS was based on the presence of behavioral problems between 3 pm and 12 am, and its severity was calculated as a product of the number of symptoms and their frequency [5]. A similar approach was implemented in a recent Italian study where the authors created a questionnaire based on the Neuropsychiatric Inventory (NPI), which is widely used in the assessment of neurobehavioral disorders in dementia [6]. The questionnaire was used to determine if behavioral worsening was related to evenings/nighttime and then to clarify the precise features of the worsening. These examples lead to the conclusion that intuitive descriptions of SS as behavioral worsening that is dependent on the time of the day are suitable for epidemiologic studies and clinical practice when they are supported by a questionnaire.

Analysis of the definitions of SS used in various studies leads to the conclusion that these definitions are rather caregiver-centered. The description of SS relies on phenotypic features (symptoms) that are problematic to the caregiver of the patient (e.g., wandering, aggression). Much less attention is given to the perspective of the patients. Their subjective fear or feeling lost are not considered in the definitions. Therefore, the following definition of SS may be coined:

1. Abrupt development of behavioral changes in a demented patient;
2. Appears during the evening or night (in the darker part of the day);
3. Consists of symptoms especially problematic to the surroundings of the patient/caregiver: aggression, wandering, agitation, severe delusions or fear.

Various approaches to the definition or diagnosis of SS are presented in Table 1.

## 3. Epidemiology of SS

The difficulties in forming a clear definition of SS complicate epidemiological studies. The literature contains very divergent information due to the different criteria adopted for diagnosing SS.

Most studies claim that it is a common problem. Physicians that regularly provide care to patients with dementia syndromes, hear complaints from caregivers or families about the nocturnal behavior of patients. Meanwhile, epidemiological studies report a prevalence of SS in 2.5% to 66% of patients with dementia syndromes. Unfortunately, there is no information on whether this phenomenon is more common in specific types of dementia (e.g., Alzheimer’s, vascular dementia, or Parkinson’s disease dementia). There are also no published data linking the occurrence of this syndrome to the sex or age of patients or the duration of the disease. In a multicenter Italian epidemiological study, sleep disorders were found in more than half of the patients with Alzheimer’s disease (AD) [7]. In 1995, Little et al. found SS in 24% of patients, while Volicer et al. recognized this phenomenon in over 40% of patients based on the collected data from the patients and their families [8,9].

More recent papers have shed more light on the epidemiology of SS and its clinical correlates. An Italian study conducted on a group of 184 patients with AD led to the observation that SS was present in 21.2% of them. The most common symptoms of SS were agitation, irritability, anxiety, and delusions. There were no statistically significant differences between “sundowners” and “non-sundowners” in terms of the sex ratio, type of dementia, or clinical phenotype of the disease (based on the symptoms present, not their severity). Sundowners were found to have more profound dementia (with worse scores in cognitive and independence/daily activity measures) [6]. An epidemiologic link between the development of SS and the presence of the ε4 allele of the apolipoprotein E (*APOE*) gene was discovered in that study. The authors also noted that SS was present in 27.8% of patients. The sundowners in this study had worse results in the Clinical Dementia Rating scale (but not in the MiniMental State Examination). Of particular interest was that the patients with SS did not differ from the non-sundowners in terms of sleep quality, except for a higher frequency of REM sleep behavior disorder (RBD) in patients experiencing SS. The latter finding could be attributed to the following facts: RBD is not a common syndrome in patients with AD; the diagnosis was not supported by polysomnographic recordings; the authors used a sleep quality questionnaire posed to the patients’ caregiver; and clinically SS-related agitation may be difficult to distinguish from motor and verbal presentations of RBD [5]. In contrast, another research group in Spain found that the prevalence of SS in their study population was similar to what was previously described (19%) but with a noticeably higher prevalence among women and in patients who were older and had more severe dementia, particularly those who reported sleep problems more frequently. Interestingly, SS was more frequently present in patients diagnosed with Lewy body disease, again suggesting there may be an overlap between the symptoms of RBD and SS that may alter the statistics [10]. Silva et al. performed a similar study on the Brazilian population of hospitalized patients with dementia and found a prevalence of SS of 14.3%, with more advanced average age of the patients and more severe cognitive deficits and depression. It must be noted that the studied population was small (n = 70), which limits the generalizability of the conclusions [11].

Authors of a more recent study from Brazil observed prevalence of nocturnal neuropsychiatric symptoms in over half of the studied population of patients with Alzheimer’s disease. It was irritability in 55.3% of cases and aggressiveness in 42.1% [12]. A review from 2022, focused on the delirium and sundwoning syndrome reported pooled prevalence of SS as 48.9% of demented subjects [13]. SS as a reason of delayed initiation of sleep increases significantly burden of the caregivers as it was shown by recent Korean study [14].

An interesting method of analyzing the epidemiology trends of SS was presented in a study focused on the frequency of SS-related internet queries. The authors found that such queries were more frequent after weekends and in the winter (a period with shorter days and exposure to less intense solar light). These data indicate that SS is more frequent and troublesome during weekends—probably due to a lack of professional support for the family caregivers during weekends. The correlation of the frequency of queries with darker months suggests that the symptoms of SS are more pronounced when the amount of light received by patients is limited, which indicates a pathological link between dysfunction of the melatonin system and the appearance of SS [15].

It should be noted that nocturnal behavioral disorders are a risk factor for the institutionalization of patients with dementia. This is due to, among other things, the increasing level of stress for people caring for a patient with dementia and symptoms of SS, as described in the study by Gallagher-Thompson et al. [16]. The occurrence of sleep disorders also had a negative impact on the mortality of patients with AD [17,18].

A well-described phenomenon is the relationship between sleep disorders and cognitive functioning. Reduced the quality of sleep or shortened sleep times led to a deterioration of cognitive functions and acceleration of the dementia process in a number of observational studies. Scarmeas et al., in their multicenter prospective study, found that the occurrence of SS was associated with a risk of faster deterioration of cognitive functions [19].

Studies focusing on the prevalence of SS provide a high range of results. Those discrepancies may result from a lack of strict diagnostic criteria, differences in the studied population (e.g., patients living in community vs. population of nursing houses), and different pathomechanisms leading to dementia (e.g., Alzheimer’s disease, Parkinson disease, or vascular dementia). The results may be biased with the co-occurrence of other sleep disorders (like obstructive sleep apnea) or mood and anxiety disorders [20]. The prevalence of SS, regardless of differences in individual studies is high. It must be remembered that it is extremely troublesome for caregivers of the patient and severely increases the probability of placing a patient in a nursing home.

## 4. Pathomechanism of SS

Nighttime behavioral disorders in dementia are a consequence of many pathological phenomena. First, it should be remembered that changes in the circadian rhythm are part of the physiological process of aging. In elderly people, the total sleep time is shortened, with an increased number of mid-night awakenings. For this reason, seniors have problems with initiating and maintaining sleep, which increases their susceptibility to sleep disorders [21].

Regardless of age-related changes in the circadian rhythm, there is evidence that the development of SS is connected to the pathology of AD at the genetic level. The presence of the ε4 allele of the *APOE* gene, due to its impact on the formation of amyloid, is a well-known significant risk factor for developing AD. It was found in a population of Korean patients with AD that carriers of the ε4 allele of the *APOE* gene were more frequent among the subjects with SS [5]. A very interesting approach (data mining) was proposed in a recent study by Zhang et al. [22]. The authors described a network of genes related both to the development of Alzheimer’s disease and to functions of melatonin (the “pro-sleep” hormone produced in the pineal gland). The two crucial genes within that network were *MMP2* and *NR3C1*, involved in neuroinflammatory processes and the structure of the extracellular matrix. The analysis suggests that there is a clinical link between the production and action of melatonin and the development of neurodegenerative disorders. Within such a paradigm, SS is no more a consequence of dementia and becomes an intrinsic clinical element of the disease [22].

The suprachiasmatic nucleus regulates the human biological clock, influenced by cholinergic impulses from the basal nucleus of Meynert. In Alzheimer’s disease (AD), neurodegeneration damages both structures and disrupts circadian rhythms [23,24]. The suprachiasmatic nucleus undergoes neuronal atrophy and neurofibrillary degeneration, with few amyloid plaques. These changes correlate with a flattened circadian rhythm, causing consistent activity levels throughout the day [23,24]. Combined with cognitive and emotional disturbances, this may underlie SS. Melatonin, essential for sleep regulation, decreases in AD due to pineal gland calcification, disrupting sleep preparation [25,26,27]. This leads to nocturnal hyperactivity, often resulting in aggression or wandering, key features of SS.

Regardless of the degeneration and dysfunction of the melatonin system, inadequate exposure to natural light may lead to the development of SS. It was shown in a recently published meta-analysis that examined the relationship between exposure to light and the presence of neurobehavioral symptoms of dementia (including sundowning) that a lack of solar light exposure as well as the dim light inside care homes may provoke anxiety, agitation, delusions, and other symptoms of SS [28].

An important factor that may affect the occurrence of SS may be the presence of primary sleep disorders. A good example is restless legs syndrome (RLS). According to epidemiological data, it may be present in about 20% of patients with dementia [29]. On the one hand, its symptoms are so difficult to describe (even for people without cognitive deficits) that a patient with dementia may not be able to name their ailments, while on the other hand, they are so unusual and bothersome that they not only prevent the patient from falling asleep but can also cause anxiety or agitation and night wandering (especially in SS). Moreover, patients with dementia who react with agitation to symptoms of RLS may receive sedative drugs (e.g., neuroleptics) that in turn may increase the severity of RLS; without a careful diagnosis, the patients and their caregivers may be trapped in a vicious circle. Richards et al. showed that a shortening of sleep time due to RLS symptoms increases the risk of SS; in addition, 79% of patients with dementia were receiving drugs that could potentially worsen RLS [30,31].

Another element that may lead to the occurrence of SS and circadian rhythm disorders, in general, is coexisting psychiatric and somatic diseases. Problems with falling asleep and evening behavioral disorders may appear in the course of unrecognized and untreated depression. It should be remembered that depression is very common among patients with neurodegenerative diseases, and its symptoms may escape the attention of the caregiver and attending physician [32]. Somatic symptoms, such as pain or nocturia, also disturb the evening rest of patients. This problem is also related to disorders caused by the drugs that the patients are taking; for example, sleep may be hindered by preparations with a cholinergic action, such as those given to treat upper respiratory tract infections. Another group of stimulants is beta-mimetics that are used in pulmonology, which can hinder falling asleep and increase the risk of SS.

An often-overlooked cause of SS is the patient’s environment and sleep conditions. Stimulating factors, such as a poorly structured day (long naps, low activity, little sunlight), an irregular sleep schedule, improper lighting, disrupted meal times, and inadequate soundproofing or darkness, can contribute to its occurrence.

Concluding, a cascade of phenomena leading to SS may be conceptualized. The genetic background of patients possessing genes responsible for the development of dementia and circadian disorders. The expression of these genes increases in older age, parallel to age-related changes in sleep quality and quantity. The development of clinical dementia causes limitations in daily activities (therefore, patients are not tired in the evenings and do not fall asleep) and a lack of criticism and obeying rules of social life. Neurodegeneration of the melatonin system blocks internal mechanisms of falling asleep. A decrease in cognitive functions causes delusional interpretation of the objects of the nearest environment, as shown in patient in dim light. So, patients are not sleepy, active, unable to understand the conventional approach to times of day (e.g., that it is not normal to start packing things late in the evening), and surrounded by things they do not understand. That is more than enough to become disoriented, agitated, terrified, and aggressive.

The pathological process leading to sundowning syndrome probably encompasses the genetic component through its relation with *APOE*, *MMP2*, and *NR3C1* genes and the biological component resulting from neurodegeneration of brain centers responsible for the control of the circadian rhythm. The clinical effect of this neurodegeneration is the lack of the organic ability to match the time of the day with the types and intensity of activities. This process may be reinforced by the suboptimal scheme of the patient’s daily activities (e.g., the lack of physical exercises during the day) and stimulating elements in the environment (e.g., too bright light in the afternoon and evening). All these elements together finally lead to the development of SS.

## 5. Diagnostic Procedures for SS

Sleep disorders, due to their association with the progression of cognitive disorders, have negative impacts on the caregiver’s condition and increased risks of institutionalization for the patient; hence, they should be monitored and diagnosed by the team (medical and nursing) that is caring for the patient with dementia. Questions about the regularity of their circadian rhythm and quality of nighttime sleep should be part of the interview conducted during each meeting with the patient and their caregiver.

It should be expected that caregivers will spontaneously provide information about circadian rhythm disorders. The patient’s report on their dementia syndrome may be unreliable due to memory disorders and difficulties in properly describing the symptoms that they are experiencing.

Diagnostic work-up of SS should rely mostly on history taking and comprehensive interview. The clinician should gain many sources of information: the patient, family caregiver, professional caregiver (e.g., nurse), other persons living together with the patient.

The diagnostic interview should begin with the most general question possible (e.g., “How does the patient sleep?”) and then it should try to specify what the potential problems are. In the first part of the interview, it is advisable to collect information that will allow for the qualification of the occurring disorders. It is necessary to ask questions about specific symptoms:

1. Does the patient have trouble falling asleep?
2. Does the patient wake up during sleep?
3. Does the patient have hallucinations?
4. When lying in bed, does the patient show excessive activity of the lower limbs? Do they report discomfort in the lower limbs (observation for RLS)?
5. Does the patient wander at night? Do they engage in inappropriate activity (e.g., packing, preparing meals, or becoming dressed)?
6. Does aggression appear (verbal or physical)?
7. Does the patient exhibit anxiety?
8. Do cognitive disorders increase in the evening (especially orientation disorders, e.g., becoming lost in their own apartment)?

Collecting this information will allow us to determine whether we are dealing with a primary sleep disorder (trouble falling asleep in the case of insomnia or discomfort in the lower limbs in the case of RLS), a circadian rhythm disorder (undertaking normal activities, such as preparing to go for a walk or starting to cook, but at an inappropriate time), or an evening occurrence or exacerbation of psychiatric symptoms (hallucinations, delusions, aggression, and/or anxiety).

When collecting interview responses about sleep disorders, attention should also be paid to external factors that may lead to the occurrence of sleep disorders. It is necessary to ask the following questions:

1. What medications is the patient currently taking?
2. What are the other co-occurring diseases?
3. What is the patient’s typical daily schedule (occurrence of daytime naps, physical activity, regular sleep and wake times)?
4. How well lit is the patient’s place (exposure to light during the day, darkening of the bedroom at night)?
5. Does the bedroom provide sufficient comfort for rest (separate, lockable patient room, isolated from sounds from outside and inside the building)?

In the patient’s assessment, attention should also be paid to the occurrence of symptoms of depression: anhedonia, abandonment of previous interests, depression/lowering of mood, uttering content that suggests a low self-esteem or suicidal intentions, and appetite disorders. Whether the sleep disorders reported by the patient and their caregivers are an element of a depressive episode should be assessed.

The diagnostic process for SS is mainly based on an interview. A physical examination is not expected to detect symptoms that could lead to the diagnosis of a circadian rhythm disorder. Additional tests are also not very useful. In the case of suspected SS, an actigraphy test may be considered. This test, which involves a continuous recording of the patient’s movements (or, more precisely, the movement of their limbs using an attached accelerometer), allows for the determination of the patient’s daily activity pattern. In the case of SS, a flattening of the daily activity pattern should be expected in the actigraphy record: the intensity of the patient’s movements in the evening and daytime will be similar. Actigraphy can also detect irregularities in the patient’s daily activity that contribute to the occurrence of evening behavioral disorders; for example, prolonged periods of rest during the day (naps) or an increase in the frequency of lower limb movements in the evening (in the case of RLS). However, the equipment for this method is not easily accessible, and a properly conducted interview will provide little additional information that could change the diagnosis. Polysomnographic tests, which are the “gold standard” in diagnosing sleep disorders, are not useful in the case of SS. A reliable polysomnographic test should be performed in a sleep laboratory, which means that a patient with cognitive disorders would have to spend the night in an unfamiliar environment—this alone is such a significant disruption of the test conditions that drawing any conclusions about the patient’s functioning at home from polysomnography will be practically impossible. Polysomnography is only justified in the case of suspected sleep disorders that could result in the following:

1. Could pose a threat to the patient (e.g., RBD);
2. Could lead to an acceleration of cognitive disorders (e.g., sleep apnea syndrome);
3. Could potentially be amenable to therapy (e.g., sleep apnea treatment).

It must be kept in mind that both actigraphy and polysomnography requires a high level of patient compliance and co-operation. That makes performing this kind of tests difficult in the population of demented subjects. Another problem related to polysomnography is that spending a night in a sleep laboratory, which would be completely unknown environment for the patient, would severely promote stress and disturbed their sleep.

## 6. Therapeutic Treatments for SS

SS can have different clinical forms and, more importantly, different causes. The treatment of behavioral disorders in the evening hours should always be aimed at its cause. Therefore, it is important to collect data that can explain the underlying mechanism of SS in patients with dementia. Therapy for SS should be personalized (taking into consideration individual features of the patients) and multimodal (using both pharmacological and non-pharmacological approaches). This mode of therapeutic approach is efficient in cases of agitation in demented patients [33].

In situations where evening behavioral disorders are accompanied by a deterioration in cognitive functioning (for example, poorer spatial orientation, losing one’s way between individual rooms), it is necessary to consider whether procognitive therapy is intensive enough. In such a situation, an increase in the dose of an acetylcholinesterase inhibitor and the addition or increase in the dose of memantine should be considered. In addition to the effect on cognitive functions, these drugs, according to some data, improve sleep architecture. Moraes et al. found that the use of donepezil leads, among other things, to an increase in the total sleep time, with an increase in the amount of REM sleep [34]. In a study conducted by Ishikawa et al., it was shown that adding memantine led to an improvement in the quality of nighttime sleep [35]. However, it should be remembered that cholinesterase inhibitors increase the amount of REM sleep and consequently increase the likelihood of REM sleep parasomnias such as nightmares or RBDs. To minimize the risk of these disorders, it is recommended to use acetylcholinesterase inhibitors in the morning hours.

If, during the interview and examination of the patient, it turns out they have a sleep disorder and other symptoms of a low mood, they should be treated first for depression, and antidepressant therapy should be initiated. It is recommended to use mood-improving drugs with a simultaneous sedative component, which has a documented positive effect on sleep. For example, doxepin can be used. In the study by Friedmann et al. [36], this drug, apart from its antidepressant effect, had a normalizing effect on behavioral disorders. Another drug that can effectively treat depression and sleep disorders in patients with cognitive disorders is clomipramine [37]. Escitalopram is another drug that is effective in the treatment of depression and behavioral disorders. Stein et al. showed a positive effect of escitalopram on sleep architecture in the course of depression [38], while Barak et al. showed that escitalopram at a dose of 10 mg is as effective in the treatment of agitation and psychotic symptoms as 1 mg of risperidone [39]. Trazodone is another drug that alleviates the symptoms of depression and leads to the disappearance of behavioral disorders (e.g., agitation) and therefore, can be used in the treatment of mood disorders associated with SS. This drug, at a dose of 50 to 250 mg per day, effectively controls the symptoms of agitation in patients with co-occurring depression [40].

SS can manifest as initiating “normal” activities at abnormal times (for example, preparing for a walk at 11:30 p.m.). Such a situation can be interpreted as a circadian rhythm disorder secondary to a neurodegenerative process. In such a case, the use of melatonin at a dose of 2 to 10 mg per day can be considered. Such a decision is theoretically justified: melatonin is a substance that controls the circadian rhythm, and its use alleviates, for example, the symptoms of “jet lag”. Data on the use of melatonin in patients with neurodegenerative diseases are conflicting. Most often, clinical studies show a subjective improvement in sleep quality (according to both the patient and caregiver), with no objective evidence of such improvements [41,42]. Regardless of sound theoretical background, trials with melatonin as a delirium-preventing drug bring negative results [43,44] while it has been proven to improve sleep alongside cognitive improvement in dementia [41]. This discrepancy suggests that nocturnal delirium/sundowning syndrome is a more complicated phenomenon than just a sleep–wake rhythm disorder. Melatonin-dependent processes are only partially responsible for preventing the development of SS.

In a recently published paper, Grippe et al. showed that therapy with trazodone can also normalize the circadian rhythm [45]. In addition to endogenous circadian rhythm disorders, it should be remembered that sleep–wake cycle disorders due to environmental factors, such as an abnormal distribution of activity during the day or night resting activity, are even more common. There are a number of possible corrective actions in this area that can bring about improvement. Observational studies have shown that implementing interventions such as daily physical activity for at least 30 min (especially outdoors), eliminating naps during the day, maintaining fixed hours of falling asleep and waking up, avoiding staying in the bedroom during the day, and creating comfortable sleeping conditions could lead to significant improvements in sleep quality [46,47].

Some promising data were recently published that pointed at potential benefits of simple behavioral interventions. Patients with dementia were asked to spend 120 min per week walking with their caregivers. After implementing this regime for 6 months, a significant improvement was noted in terms of the frequency and intensity of their SS symptoms, especially in those subjects who took their walks in the afternoon [48].

There is some evidence that environmental interventions may have a positive impact on SS and sleep–wake rhythm disorders in neurodegenerative diseases. A 2 h session of additional morning lighting performed over a period of 4 weeks resulted in significant improvement in the quality and duration of sleep [49]. Using blue-enriched lighting in care homes led to a reduction in anxiety which may reduce the frequency of SS [50]. Other potentially effective environmental and behavioral interventions include music therapy, activity-based therapy, and usage of virtual reality [33].

In some cases, SS may be a consequence of insomnia, i.e., difficulty in initiating sleep. In such situations, the use of hypnotics, i.e., benzodiazepines or gamma-aminobutyric acid (GABA) receptor agonists (so-called “Z-drugs”), is not recommended. Firstly, these drugs are recommended for the short-term treatment of insomnia, and neurodegenerative diseases are a chronic problem. Secondly, a number of the adverse effects associated with these drugs may worsen the condition of patients with dementia. In the case of insomnia, the introduction of antidepressants, such as doxepin, should be considered. A recently published study showed that the use of doxepin did not significantly affect the balance of disorders and the risk of falls when waking up at night, which speaks to the safety of this drug [51]. Trazodone has been repeatedly tested in the treatment of insomnia (both in the course of depression and without mood disorders). The summary of the available clinical trials showed that this drug can be considered (initially in a small dose) in the treatment of insomnia and in patients with cognitive disorders [52]. The most serious element of SS is the presence of psychotic symptoms (delusions and hallucinations) and agitation with aggression. Unfortunately, the therapeutic options are limited here. Nevertheless, several studies have shown that second-generation neuroleptics, such as olanzapine, are effective in reducing aggression and agitation in patients [53]. The use of these drugs also has a positive effect on the level of burden felt by caregivers of patients [54].

It must be remembered that pharmacotherapy, although very temptative due to the ease of its use and its effectiveness, has its limitations. Neuroleptics, Z-drugs, bezodiazepines lead to a decrease in attention during the day, therefore they may exacerbate cognitive disorders. Daytime sleepiness which is another consequence of these drugs, limits the activity of the patient during the day. As a result patient is less tired and sleepy in the evening which increases the probability of developing SS. Repetitive solving problem of SS with sedative drugs will close the patient and the caregiver in a vicious circle leading to increasing doses of sedatives and their limited usefulness. As shown in a retrospective analysis of over 27,000 demented British patients, usage of Z-drugs was related to increased risk of falls, fractures, and strokes [55]. Sedative drugs (including antidepressants, antipsychotics or anxiolytics) were found to increase the risk of falls and delirium in another study [56]. It is also suggested by some studies use of sedatives may lead to cognitive decline [57]. A physician deciding to start treating SS with sedative drugs must be aware of these threats and perform risk–benefit analysis.

Behavioral interventions are free of such threats and are extremely resource consuming. Activity-based interventions (e.g., long walks every day), strict supervision over the patient’s timetable (e.g., repetitive hours of waking and going to bed), or elimination of daytime naps require the involvement of the caregivers (members of family or nursing staff); therefore, are difficult and expensive. Meta-analytic studies show that behavioral interventions in a demented subject are more efficacious than pharmacologic ones [58]. Such solutions are cost-effective as well as require enormous involvement of family and staff [59]. It is probable that the solution lies in a personalized, patient-centered approach with various therapeutic modalities used simultaneously with doses/intensity dependent on the patient’s individual features.

## 7. Summary

Table 2 presents a simplified summary of the possible clinical phenotypes of sundowning syndrome (SS) and possible therapeutic interventions. In practice, there is rarely a single explanation for the observed symptoms. Therefore, SS is a clinical phenomenon that eludes clear categorizations. What is crucial about this syndrome is its close pathological relation to neurodegenerative process. Therefore, further studies on SS are likely to shed new light on the role of circadian rhythm and its disorders in pathogenesis of dementia. On the other hand, clinical trials in Alzheimer’s disease and other neurodegenerative disorders should take into consideration the impact of tested drug on SS. Evening behavioral disorders in the course of dementia are a serious problem, especially for the patient’s caregivers. It should be remembered that there is no single, universal therapeutic method. The diagnostic process should lead to the identification of the factors provoking the patient’s evening deterioration in functioning, which will allow for the appropriate selection of a therapeutic intervention. The therapeutic intervention should be tailored to dominant problems of the patient and to diagnosed causes of SS. Sleep hygiene will be effective in patients with disordered circadian rhythm of activities or with insomnia. Aggressive or hallucinating patients should receive neuroleptic treatment. Demented patients with irritability or mood disorders as well as the ones with chronic insomnia should benefit from antidepressant therapy.

In conclusion, it must be said that SS is a frequent although not well-defined problem in patients with dementia. Its etiology is related to a neurodegenerative process, patient’s co-morbidity, behavior, and environment. Diagnosis should be based mainly on history and physical examination. Therapy should address the suspected triggering factor of SS and the most troublesome symptoms of SS. The most effective should be a multi-modal personalized approach including pharmacological and non-pharmacological interventions.

## Figures and Tables

**Table 1:** Various approaches to the definition of sundowning syndrome.

Mode of Definition	Description	Author
Presence of agitation	The presence of agitation during the evening and night. Symptoms of agitation were listed accordingly to Cohen–Mansfield Agitation Inventory.	Shih et al. (2017) [4]
Specific questionnaire	Sundown Syndrome Questionnaire (SSQ): the caregiver is asked to point at agitation symptoms that appear in the patient, with denoting time of the day they are present and their weekly frequency. SSQ allows also measurement of severity of the SS by multiplying the number of symptoms by the frequency of their occurrence.	Pyun et al. (2019) [5]
Caregiver questionnaire	A questionnaire filled in by a caregiver. A screening question was focused on the presence of any behavioral change in the patient’s behavior in the evening hours. If answer was positive then the caregiver was asked to select symptoms appearing in the evening from a list based upon Neuropsychiatric Inventory (NPI).	Toccaceli et al. (2023) [6]

**Table 2:** Possible clinical phenotypes of SS and proposed therapeutic interventions.

Clinical Phenotype	Proposed Intervention
Poor sleep hygiene; Daytime naps; No fixed bedtimes; Poor sleep environment.	Implementation of sleep hygiene rules.
Evening deterioration of cognitive functioning; Disorientation; Wandering.	Optimizing procognitive therapy.
Low mood; Insomnia; Irritability; Diagnosis of depression.	Initiation of an antidepressant with a sedative effect (doxepin, trazodone, escitalopram).
Undertaking “normal” activity at an “abnormal” time; Circadian rhythm disorders.	Implementation of sleep hygiene rules; Adding melatonin; Possible positive effect of trazodone.
Difficulty in initiating sleep; Insomnia.	Behavioral interventions (sleep hygiene); Antidepressant drug therapy (doxepin, trazodone).
Aggression; Agitation; Psychotic symptoms.	Adding sedative drugs; for example, neuroleptics.

## Abbreviations

The following abbreviations are used in this manuscript:

SS	Sundowning syndrome
AD	Alzheimer’s disease
RBD	REM sleep behavior disorder
RLS	Restless legs syndrome
